# Safety and Tolerability of Initiating Maximum-Dose Sacubitril-Valsartan in Patients on Target Dose Renin-Angiotensin System Inhibitors

**DOI:** 10.1155/2019/6745074

**Published:** 2019-08-01

**Authors:** Helena Norberg, Ellinor Bergdahl, Krister Lindmark

**Affiliations:** ^1^Department of Public Health and Clinical Medicine, Umeå University, 901 87 Umeå, Sweden; ^2^Department of Pharmacology and Clinical Neuroscience, Umeå University, 901 87 Umeå, Sweden

## Abstract

**Aim:**

Sacubitril-valsartan has proven beneficial in heart failure with reduced ejection fraction. Guidelines recommend initiating half-dose sacubitril-valsartan before up-titration even to patients already on target dose angiotensin-converting enzyme (ACE) inhibitors or angiotensin receptor blockers (ARB). To reduce the number of titration steps needed in order to simplify for the patient as well as the clinic, we aimed to investigate the safety and tolerability of switching patients on target dose ACE inhibitors or ARBs directly to maximum-dose sacubitril-valsartan.

**Methods:**

This prospective cohort study was conducted between April 2016 and November 2017. A total of 66 patients with heart failure and reduced ejection fraction already on guideline-recommended target dose ACE inhibitors or ARBs (equivalent to enalapril 10 mg twice daily) were switched to maximum-dose sacubitril-valsartan (200 mg twice daily). The patients were followed for twelve months.

**Results:**

Patients had a mean age of 72 ± 10 years, mean systolic blood pressure of 121 ± 17 mmHg, and 92% were male. At 12-month follow-up, nine patients (14%) had discontinued sacubitril-valsartan, four patients (6%) had a dose reduction, and 17 patients (26%) had developed symptomatic hypotension. No angioedema occurred within the 12-month follow-up and there were no hospitalizations or emergency room visits within the first 14 days.

**Conclusions:**

Switching directly from target dose ACE inhibitors or ARBs to maximum-dose sacubitril-valsartan was safe and generally well tolerated.

## 1. Introduction

Despite advances in treatment, heart failure (HF) remains one of the leading causes of hospitalization, morbidity, and mortality [1, 2]. In addition to the consequences for the individual patient, HF is a vast economic burden to the healthcare systems [2].

Sacubitril-valsartan was approved by the U.S. Food and Drug Administration (FDA) and European Medicines Agency (EMA) in 2015, based on the randomised controlled PARADIGM-HF trial (Prospective Comparison of Angiotensin Receptor-Neprilysin Inhibitor with angiotensin-converting enzyme (ACE) inhibitor to Determine Impact on Global Mortality and Morbidity in HF) [3]. In the PARADIGM-HF study, patients who had been pretreated with an ACE inhibitor or angiotensin receptor blocker (ARB) in a dose equivalent to minimum 10 mg enalapril daily first underwent a run-in period of up-titration to target dose enalapril (10 mg twice daily) for two weeks. Patients without unacceptable adverse reactions were then switched to sacubitril-valsartan (starting dose 100 mg twice daily, which was doubled to maximum-dose 200 mg twice daily) during four to six weeks. To reduce the risk of angioedema, a wash-out period of 36 hours was applied between the last dose of ACE inhibitor and initiation of sacubitril-valsartan. The PARADIGM-HF trial showed a 20% reduction in cardiovascular mortality and HF hospitalizations in the sacubitril-valsartan group compared to the enalapril group [3]; however, subsequent studies have shown a slow adoption of sacubitril-valsartan in real-world patients [4, 5].

HF guidelines recommend several titration steps with ACE inhibitors/ARBs as well as with beta-blockers, followed by evaluation for mineralocorticoid receptor antagonists (MRA) [6, 7]. If the patient is still symptomatic and has a low ejection fraction, subsequent titration steps with sacubitril-valsartan should follow. With so many titration steps, there is a risk for patient fatigue which could lead to nonadherence [8, 9]. There is also a risk of stressing the resources of the individual clinic or clinician [8, 9], especially with a novel treatment such as sacubitril-valsartan where there may be a large patient population who suddenly is eligible for a new treatment. There is clearly a need for methods to simplify the implementation of novel therapies, both for the patients and the clinics, and to reduce the number of necessary titration steps.

The aim of this study was to investigate the safety and tolerability in switching patients on target dose ACE inhibitors/ARBs directly to maximum-dose sacubitril-valsartan.

## 2. Methods

### 2.1. Participants and Study Design

This prospective cohort study was conducted at the Umeå University Hospital, Sweden. Patients were included between April 2016 and November 2017. In total, 1924 patients with an HF diagnosis (according to the* International Classification of Diseases, 10th revision *codes I50.X, I.42X, I11.0) were screened for eligibility for sacubitril-valsartan, which has previously been described [10]. To be eligible for sacubitril-valsartan, patients needed to fulfill the main entry criteria from the PARADIGM-HF trial: 18 years and older, EF ≤ 35%, N-terminal pro-B-type natriuretic peptide (NT-proBNP) ≥ 600 pg/mL, estimated glomerular filtration rate ≥ 30 mL/min, systolic blood pressure ≥ 95 mmHg, and serum potassium level < 5.4 mmol/L at their latest sampling. We only included patients on target dose ACE inhibitors/ARBs, defined as the target dose recommended in the European Society of Cardiology guidelines [6]. Eligible patients who were identified through the screening process or who became eligible during the study period were offered sacubitril-valsartan. Patients who initiated the treatment were asked to participate in the follow-up study.

At the baseline visit, we performed routine laboratory tests, including NT-proBNP, blood pressure measurements, and assessment for New York Heart Association (NYHA) class. To further simplify for the patients, we also attempted a 24-hour wash-out period for ACE inhibitors instead of the recommended 36-hour period [6, 7]. The same procedure was used for ARBs. Patients were instructed on possible adverse events and were asked to contact the clinic if they experienced any problems after commencing treatment. The patients were also instructed that sacubitril-valsartan has a diuretic effect and that they should try to reduce the dose of loop-diuretics.

Patients were followed up with blood pressure measurements after 2 weeks if systolic blood pressure was 110 mmHg or less at the baseline visit. Further, a telephone follow-up of adverse events was performed after 3 months in patients who did not have any other scheduled visit within this time period. One year after sacubitril-valsartan was initiated, patients were summoned to a return visit. At the 12-month follow-up, we performed a clinical evaluation and recorded any treatment change, reasons for change, as well as evaluated patients' clinical status with blood pressure measurements and routine laboratory tests.

Tolerability was assessed as patient-reported adverse events, need of dose reduction, and treatment discontinuation. Safety was assessed as occurrence of hospitalizations or emergency room visits within 14 days of initiation or development of angioedema within 12-month follow-up.

### 2.2. Statistical Analysis

Normally distributed continuous variables are reported as means with standard deviations and nonnormally distributed continuous variables as medians with interquartile range. Categorical variables are described as frequencies with percentages. Comparisons between baseline and follow-up were made with paired t-test. We performed all analyses with IBM SPSS Statistics, Version 25.0 (Armonk, NY: IBM Corp.).

## 3. Results

A total of 66 patients were included in the study between April 2016 and November 2017. Baseline characteristics are displayed in Table 1. The group was predominately male (92%), white (98%), with a mean age of 72 ± 10 years, and mainly belonged to NYHA class II or III (29% and 61%, respectively). Mean systolic blood pressure was 121 ± 17 mmHg with four patients having a systolic blood pressure of 95 mmHg.

**Figure 1:**
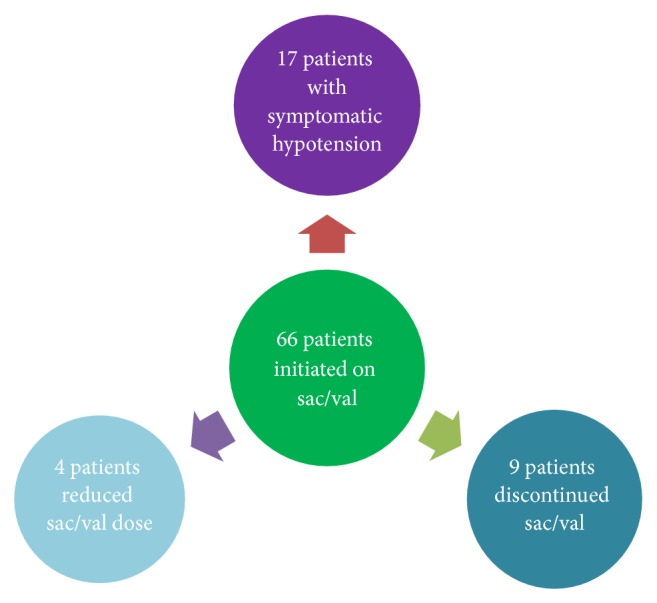
Overview of patients initiated on sacubitril-valsartan (sac/val).

**Figure 2:**
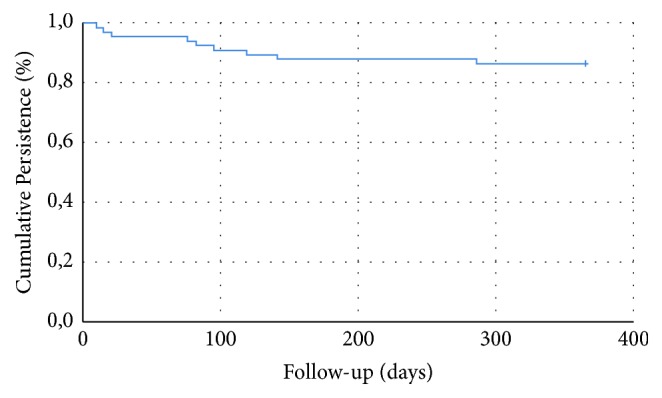
Kaplan-Meier curve of persistence on sacubitril-valsartan.

An overview of patients who initiated sacubitril-valsartan is shown in Figure 1. Four patients (6%) had to reduce the sacubitril-valsartan dose within the first year. A total of nine patients (14%) discontinued treatment, eight of them (12%) due to adverse events. The earliest treatment termination was after 10 days; otherwise, treatment was suspended after a median of three months (see Figure 2). The most common reasons for discontinuation were a slowly developing itching rash during the first weeks after initiation (*n* = 3, two patients with previous ACE inhibitor therapy and one patient with previous ARB) and progressive renal failure (*n* = 2), both of which were switched back to ACE-I or ARB instead of lowering the dose of sacubitril-valsartan. Another 17 patients (26%) reported symptomatic hypotension during the first year of treatment, of which one patient discontinued treatment, three patients reduced the dose of sacubitril-valsartan, and thirteen were able to remain on the same dose. All reasons for discontinuation are presented in Table 2. Three patients died during follow-up, none of them during the first three months of treatment. There were no occurrences of hospitalizations or emergency room visits within the first 14 days or angioedema during the 12-month follow-up. There were no significant differences between baseline, three month follow-up, and one-year follow-up with regard to serum creatinine, serum potassium, and NT-proBNP. Blood pressure was not measured at three months. Systolic blood pressure was significantly lower at one-year follow-up (121 ± 17 mmHg at baseline vs. 115 ± 15 mmHg, paired t-test p<0.05). There was no significant difference in diastolic blood pressure at one-year follow-up.

Before switching to sacubitril-valsartan, all patients were treated with guideline-recommended target doses of ACE inhibitor (*n* = 37) or ARB (*n* = 29). Two patients had a combination of target dose ARB and a low dose ACE inhibitor. The ACE inhibitor group was either treated with enalapril 20 mg daily (*n* = 20) or ramipril 10 mg daily (*n* = 17). The ARB group was treated with candesartan 32 mg daily (*n* = 20) or losartan 150 mg daily (*n* = 9). Of the 37 patients that switched from ACE inhibitors, 36 patients waited 24 hours between last-dose ACE inhibitor and the first-dose sacubitril-valsartan and one patient waited 48 hours.

A total of 47 patients (71%) were treated with MRA at baseline. At one-year follow-up, three patients had to discontinue treatment, two of which had already had to discontinue treatment with sacubitril-valsartan and the third patient discontinued treatment shortly before death. One patient had his dose of MRA reduced and three patients had their dose increased. One patient who did not receive treatment with MRA at baseline was successfully reintroduced on treatment during follow-up.

## 4. Discussion

Initiating maximum-dose sacubitril-valsartan in patients tolerating target dose ACE inhibitor/ARB was safe and did not result in any early hospitalizations or emergency room visits. Sacubitril-valsartan was generally well tolerated in this cohort with 12% of the patients discontinuing treatment due to adverse reactions within the first year. These results are in line with the PARADIGM-HF study where 11% of the patients discontinued sacubitril-valsartan because of an adverse event [3]. Symptomatic hypotension was more common with 26% in our cohort compared to 18% in the PARADIGM-HF trial [11, 12]. However, in the PARADIGM-HF trial, there was a run-in phase where patients were excluded if they experienced significant hypotension which makes the comparison difficult. Even if there were a significant number of patients who experienced hypotension in our study, this was mostly mild, and a majority did not have to reduce the dose of sacubitril-valsartan.

European and American guidelines [6, 7], as well as the prescribing information [11, 12], recommend a starting dose of sacubitril-valsartan (100 mg twice daily for 2-4 weeks) before the maximum-dose (200 mg twice daily) is initiated. This is actually a down-titration of active drug from target dose ACE inhibitor/ARB to half-dose valsartan. Switching directly from target dose ACE inhibitor/ARB to maximum-dose sacubitril-valsartan ensures that the patients do not receive a suboptimal dose of ARB during the titration phase. In addition to the presumed benefit for the patient to keep full dose of renin-angiotensin system blockade, there is also an advantage for the clinic that initiates treatment since switching directly to maximum-dose sacubitril-valsartan reduces the number of follow-up visits.

The number of included patients may seem low as we screened 1924 patients with HF. However, for budget reasons, we reasoned with the local pharmacological committee and agreed to only include patients who would fulfill the strict inclusion criteria in the PARADIGM-HF study. As we have previously shown, this only applies to around 5% of our total HF population and approximately 25% of the HF population with reduced ejection fraction [10]. In that paper, we identified 95 patients who would be eligible for treatment, but as some lost eligibility, died, or were unfit to participate in this follow-up study, we were still able to include a majority of patients who were eligible for treatment.

Studies have reported a slow adoption of sacubitril-valsartan in clinical practice [4, 5]. Contributing factors to the slow implementation have been suggested as high cost, patient access to the medication, and delay in new guidelines. A retrospective cohort study also showed that one-third of patients who initiated sacubitril-valsartan in the U.S. were nonadherent to treatment within the first 180 days [5]. Patients with chronic obstructive pulmonary disease, patients residing in the South, and black patients had a poorer adherence, while patients previously on ACE inhibitors/ARBs and patients initiated on maximum-dose sacubitril-valsartan had better adherence. In addition, another study has shown that up-titration is often not attempted during the first six months in patients initiated on lower doses of sacubitril-valsartan [13]. This indicates that for patients who are likely to tolerate higher doses,* e.g*., patient already on target dose ACE inhibitor/ARB, a direct switch to maximum-dose sacubitril-valsartan would be preferred.

With the introduction of a treatment that lowers blood pressure, there is a concern that other treatments must be discontinued. We did not notice any such tendencies with regard to MRA as two of the three that were discontinued on MRA earlier had to terminate sacubitril-valsartan and the third discontinued use in the palliative stage of the disease shortly before death.

Our experience was that patients who had a moderately low blood pressure from the beginning, even with systolic blood pressure slightly below 100 mmHg, tolerated the switch to maximum-dose sacubitril-valsartan. However, patients with low blood pressure who already had problems with symptomatic hypotension were generally reluctant to try this treatment approach and were not included in the study. In the PARADIGM-HF trial, 95 mmHg was the limit for systolic blood pressure at randomization. European and American guidelines recommend to observe for hypotensive symptoms and only to initiate sacubitril-valsartan to patients with adequate blood pressure [6, 7]. Hence, we would still advice caution in patients with low blood pressure before attempting to switch to full-dose sacubitril-valsartan [14].

Further, our population had a higher proportion of patients with symptomatic hypotension compared to the PARADIGM-HF trial. On the other hand, the number of adverse events would be expected to be higher in real-world populations than reported in the clinical trial owing to the applied run-in period where patients that developed adverse reactions during the first weeks of treatment were excluded before randomization. Our patients were also older than in the PARADIGM-HF study (mean age 72 years* vs.* 64 years) indicating an increased risk of adverse events.

With nine patients discontinuing treatment within the first year, we did not expect that a third of them would need to terminate their medication due to an itching rash that slowly developed during the first weeks of treatment. This adverse event seemed rare in the PARADIGM-HF study [3], which could possibly be explained by the exclusion of patients with adverse reactions during the run-in period. Another contributing factor could be an increase in bradykinin concentrations owing to our abbreviated wash-out period of 24 hours instead of the guideline-recommended 36 hours. However, this seems unlikely because one of the patients was pretreated with an ARB instead of an ACE inhibitor and none of the patients experienced a rapid-onset rash.

We also attempted a 24-hour wash-out period with ACE inhibitors instead of the recommended 36-hours. ACE inhibitors together with sacubitril increase the risk for angioedema which is a serious but rare adverse event. Sacubitril-valsartan alone has shown a frequency of angioedema of < 0.5% [15] and higher rates in black patients. We did not have any cases of angioedema. It should be noted that we included a limited number of mainly white patients who tolerated target doses of ACE inhibitors/ARBs and this patient group has a low absolute risk to develop angioedema. Sacubitril-valsartan has, however, been approved for more than two years without any major safety concerns [16–19], and the risk for angioedema seem to be low also in the real world so far. To be on the safe side, for both angioedema and itching rash, a minimum of 36-hour wash-out could be used especially in black patients and patients with impaired renal function that have prolonged half-life of the ACE inhibitor.

We only studied patients already on target dose ACE inhibitor/ARB. If one chooses to switch to sacubitril-valsartan in patients on lower doses of these medications, more caution is advised and a lower starting dose of sacubitril-valsartan is recommended [6, 7]. According to guidelines, it is recommended to try to up-titrate standard therapy first, but a considerable proportion of the HF population does not tolerate target doses of ACE inhibitors/ARBs [10, 20–22]. In the future, sacubitril-valsartan may prove superior to ACE inhibitors/ARBs at new-onset HF if ongoing studies show benefit [23, 24]. If this will be the case, the need to first titrate ACE inhibitor/ARB and commencing MRA before switching to sacubitril-valsartan may be obsolete. This will simplify matters even more. However, as ACE inhibitors/ARBs remain a low-cost alternative they may remain as first-line treatment in some countries for budget reasons. Additionally, the large patient groups already on ACE inhibitors/ARBs with indication for sacubitril-valsartan still need to do the switch.

### 4.1. Limitations

The small sample size and the single-centre study design limit the generalizability and external validity of the results. However, our patients were older than in the PARADIGM-HF study and probably reflect the real-world HF population more accurately. Further, the incidence of angioedema was 0.2 % in the PARADIGM-HF trial and this study was underpowered to assess safety with the 24-hour wash-out period. Additionally, the follow-up procedure may differ from the standard of care in other HF clinics, which could have underestimated the number of patient-reported adverse events in our study. We tried to reduce the risk of underestimation by encouraging patients to contact us if they experienced any inconvenience after sacubitril-valsartan initiation also between the scheduled follow-up visits.

## 5. Conclusion

Switching directly from target dose ACE inhibitors or ARBs to maximum-dose sacubitril-valsartan was safe and generally well tolerated.

## Figures and Tables

**Table 1:** Baseline characteristics.

Characteristics*∗*	Patients (*n* = 66)
Age, years	72 ± 10
Male sex (*n*, %)	61 (92)
Race or ethnic group (*n*, %)	
White	65 (98)
Black	1 (2)
Systolic blood pressure, mmHg	121 ± 17
Diastolic blood pressure, mmHg	72 ± 11
Ejection fraction, %	30 ± 6
Heart rate, beats/min	76 ± 18
Serum potassium, mmol/L	4.4 ± 0.4
Serum creatinine, *μ*mol/L	107 ± 24
Creatinine clearance, ml/min	76 ± 32
Body weight, kg	93 ± 21
BMI, kg/m^2^	31 ± 10
NT-proBNP, ng/L (median, IQR)	1612 (774-3515)
NYHA class (*n*, %)	
I	2 (3)
II	19 (29)
III	40 (61)
IV	5 (7)
Medical history (*n*, %)	
Hypertension	42 (64)
Diabetes	19 (29)
Coronary artery disease	37 (56)
Atrial fibrillation	35 (53)
Medications (*n*, %)	
ACE inhibitor*∗∗*	37 (56)
ARB*∗∗*	29 (59)
Beta-blocker	63 (95)
MRA	47 (71)
Diuretics	42 (64)

ACE, angiotensin-converting enzyme; ARB, angiotensin receptor blocker; BMI, body mass index; IQR, Interquartile range; MRA, mineralocorticoid receptor antagonist; NT-proBNP, N-terminal pro-B-type natriuretic peptide; and NYHA, New York Heart Association.

*∗*All values were reported as mean ± standard deviation unless otherwise indicated.

*∗∗*All patients were prescribed ACE inhibitors or ARBs in doses equivalent to enalapril 20 mg daily.

**Table 2:** Reasons for discontinuation of sacubitril-valsartan within first year.

Number	Patient	Reason for discontinuation
1	*87-year-old man*	Dizziness, syncope, and a slowly developing itching rash
2	*82-year-old woman*	Irritated bowels
3	*81-year-old man*	Slowly developing itching rash
4	*80-year-old man*	Progressive kidney failure
5	*79-year-old man*	Orthostatic hypotension, even after dose reduction
6	*76-year-old man*	Coughing
7	*77-year-old man*	Progressive kidney failure
8	*76-year-old man*	Slowly developing itching rash
*9*	*69-year-old man*	Decided he did not want to continue with medication that is not currently endorsed by national guidelines

## Data Availability

The individual patient data used to support the findings of this study are available from the corresponding author upon request.
